# Development of Fermentation Strategies for Quality Mild Coffee Production (*Coffea arabica* L.) Based on Oxygen Availability and Processing Time

**DOI:** 10.3390/foods14173001

**Published:** 2025-08-27

**Authors:** Aida Esther Peñuela-Martínez, Carol Vanessa Osorio-Giraldo, Camila Buitrago-Zuluaga, Rubén Darío Medina-Rivera

**Affiliations:** National Coffee Research Center, Cenicafé 170009, Colombia

**Keywords:** physical and sensorial coffee quality, microbial shift conditions, embryo viability, semianaerobic fermentation, self-induced anaerobic fermentation

## Abstract

Fermentation is a crucial stage in the production of washed mild coffees, as it enables the generation of compounds that influence overall quality. The conditions to optimize this process are still unknown. This study evaluated the effects of fermenting coffee fruits and depulped coffee under two conditions: an open tank (semi-anaerobic-SA) and a closed tank (self-induced anaerobic fermentation, SIAF) over 192 h. Samples were taken every 24 h using a sacrificial bioreactor. A randomized complete block design with a factorial arrangement (2 × 2 + 1), plus a standard control, was employed, incorporating two factors: coffee type and fermentation condition. High-throughput sequencing of 16S and ITS amplicons identified an average of 260 ± 71 and 101 ± 24 OTUs, respectively. *Weisella* was the dominant lactic acid bacteria, followed by *Leuconostoc* and *Lactiplantibacillus*. Acetic acid bacteria, mainly *Acetobacter*, were more abundant under semi-anaerobic conditions. The yeast genera most affected by the fermentation condition were *Pichia*, *Issatchenkia*, and *Wickerhamomyces*. Repeated measures analysis revealed significant differences in pH, glucose consumption, lactic acid production, dry matter content, embryo viability, and the percentage of healthy beans. Principal component analysis was used to develop an index that integrates physical, physiological, and sensory quality variables, thereby clarifying the impact of each treatment. Samples from shorter fermentation times and SIAF conditions scored closest to 1.0, reflecting the most favorable outcomes. Otherwise, samples from longer fermentation times in both depulped and coffee fruits scored 0.497 and 0.369, respectively, on the SA condition. These findings support technically and economically beneficial fermentation strategies.

## 1. Introduction

Post-harvest processing is widely recognized as a key factor influencing coffee quality, largely due to its inherently multifactorial nature [1,2,3,4,5]. As a result, coffees processed using dry, semi-dry, or wet methods often exhibit distinct differences in their perceived attributes [4,6,7]. These methods differ based on which part of the fruit is used to reduce moisture content and produce green coffee—whether it is the whole fruit, depulped beans, or washed beans—resulting in natural, honey, or washed mild coffees, respectively. Given fermentation’s potential to drive biochemical transformations, various approaches are now being explored to influence this process and shape the sensory profile of the final product [7,8,9,10].

Traditional wet fermentation is primarily used to remove the mucilage naturally. In coffee processing, fermentation refers to the breakdown of complex carbohydrates by the native microbiota after the exocarp has been removed through depulping [7,11,12]. This stage is typically described as aerobic, semi-anaerobic, or anaerobic (including self-induced anaerobic fermentation, or SIAF), depending on the conditions under which it takes place. Fermentation is considered aerobic when the coffee is spread in thin layers on platforms or patios, exposing it to air. This allows mucilage to break down while also reducing moisture content and is used for both whole fruits and depulped beans, in the production of natural and honey coffees. This method allows for spontaneous fermentation and may also involve inoculation with starter cultures sprayed onto the coffee [7,9]. Semi-anaerobic fermentation involves using open containers, allowing microorganisms to consume the oxygen available between the beans. This creates microaerobic conditions at different depths of the coffee mass, encouraging continuous microbial activity. It can be conducted with or without water, and either spontaneously or using selected microbial strains [9,11,13]. In anaerobic fermentation, the process takes place in sealed containers—either with carbon dioxide injection [4,14] or through the SIAF method, in which microorganisms consume the available oxygen as part of their metabolism, releasing CO_2_ and other volatile and non-volatile compounds that alter the environment inside the tank. This promotes the activity of facultative anaerobes like yeasts and lactic acid bacteria [15,16] shifting the fermentation pathway and offering greater control over the process. This approach was first applied to coffee by da Mota et al. (2020) [17] and Martins et al., 2020 [18] in dry and semi-dry processing. It was later evaluated in wet processing as well [19,20,21], with positive effects on aroma and an enhanced expression of fruity notes.

The chemical composition of the fruit’s exocarp includes carbohydrates, phenolic compounds, lipids, and other substances. It also contains part of the mesocarp, which contributes a significant amount of water, along with various polysaccharides such as pectin and simple sugars like glucose and fructose [22,23,24]. These compounds play an important role in shaping coffee attributes, including flavor, aroma, sweetness, and acidity. Keeping the exocarp and mesocarp in contact with the bean for extended periods—during fermentation in SIAF tanks and before drying—has been explored as a strategy to modify the bean’s chemical composition and enhance coffee quality, especially in the production of natural and honey coffees [25]. Most studies have extended fermentation up to 96 h, observing significant shifts in microbial communities, increased production of organic acids, and changes in coffee attributes [6,8,10,26]. However, little is known about the effects of extending fermentation beyond this period under varying levels of oxygen availability, or what happens if the coffee is then depulped and processed using the wet method to produce washed coffee. It is assumed that overly long fermentation can lead to undesirable traits—such as off-flavors or astringency—especially if the process is not closely monitored. For research, it is a challenge to verify how a process that occurs in the mucilage can positively affect the quality of the coffee beverage. However, a combination of coffee type, fermentation conditions, and processing time may mark a turning point toward positive quality changes. It can serve as a starting point for identifying the optimal processing conditions.

Coffee quality—particularly in terms of attributes and sensory profile—has been widely studied in connection with fermentation [6,7,8,9,10,11,14,15,16,17,18,19,27,28,29]. Yet, other aspects that contribute to a more comprehensive understanding of quality have not yet been fully considered. This gap in knowledge may carry implications for the coffee market and economic consequences for producers, as the physical appearance of the beans serves as the initial basis for quality assessment.

To explore how substrate type and oxygen availability influence coffee quality, this study examined the effects of using whole fruits and depulped coffee under two oxygen conditions, with fermentation extended over prolonged periods. The hypothesis was that these variables could significantly affect both the physical and sensory quality of washed mild coffee. The objective of this research was to identify the most effective combination of conditions to achieve the highest possible quality in each case.

## 2. Materials and Methods

This research was conducted at the Postharvest Laboratory of the National Coffee Research Center, Cenicafé, Colombia. Ripe coffee fruits of the Castillo variety (*Coffea arabica* L.) were hand-harvested at the Naranjal Experimental Station, located in Caldas, Colombia, at an elevation of 1381 m above sea level. The site’s average temperature is 20.6 °C, with a relative humidity of 83.3% (4°58′ N, 75°39′ W).

### 2.1. Coffee Processing

Two stages of coffee—whole fruits and pulped beans—were used. Both underwent wet processing, which included sorting and cleaning by flotation in clean water, mechanical removal of the exocarp (peel), washing, and mechanical drying. Drying was performed using air at 40 °C until the parchment coffee reached a moisture content of 11 ± 0.5% w.b. Each coffee stage was subjected to two fermentation conditions: semi-anaerobic (open tank) [13] and self-induced anaerobic fermentation (SIAF) using sealed hermetic tanks equipped with an airlock valve [16,19]. For each stage and fermentation condition, eight high-density polyethylene bioreactors (30 L capacity) were used, resulting in eight bioreactors per treatment. Sampling was carried out by sacrificing one bioreactor every 24 h over a total period of 192 h.

After pulping to remove the exocarp, mucilage was removed from the coffee fruit treatments using enzymatic hydrolysis with commercial pectinlyase (EC 4.2.2.1.0) (Bogotá, Colombia) [30], to prevent interference with fermentation following pulping. Traditional fermentation using the Fermaestro^®^ method (Bogotá. Colombia) [31] was included as a control treatment. In total, five treatments were applied: pulped coffee under semi-anaerobic (SA) and SIAF conditions, whole coffee fruits under semi-anaerobic and SIAF conditions, and the control. All fermentations were conducted simultaneously in a temperature-controlled room maintained at 20 ± 1 °C.

### 2.2. Monitoring of Fermentation Processes

During fermentation, pH was monitored using a wireless pH meter with automatic temperature compensation (HI 10532/Halo^®^, Romania). The device was calibrated before each use with pH 7.0 and pH 4.0 buffer solutions (HI 7004L/C; HI 7007L/C; HANNA^®^, Romania). pH readings were taken by immersing the electrode directly into the coffee fermentation mass at each sampling point, accordingly to the procedure used for Peñuela et al., 2023a [32], and the sample value was recorded as the average of three measurements.

The temperature was continuously recorded using USB dataloggers (Uni-T UT330A), (Guangdong, China) placed at the center of the coffee mass. In addition, the concentrations of glucose and lactic acid in the mucilage were measured three times per sample using the reflectometric method with Reflectoquant RQflex^®^20 test strips (Merck SA, Darmstadt, Germany), following the procedure described in Peñuela et al., 2023a [32].

### 2.3. Microbiological Analysis

Genomic DNA (gDNA) from samples taken in triplicate at 48, 96, 144, and 192 h for each treatment was used for metataxonomic analysis. Amplicon sequencing of the bacterial 16S rRNA gene and the fungal ITS region was performed using the Illumina MiSeq platform. DNA was extracted using the commercial DNeasy PowerLyzer PowerSoil Kit (QIAGEN, Hilden, Germany), following the manufacturer’s instructions [33]. Sequencing was carried out by Macrogen Inc. (Seoul, Republic of Korea). For bacterial community profiling, a fragment spanning the V3–V4 variable regions of the 16S rRNA gene was amplified using the primers Bakt_341F (5′-CCTACGGGNGGCWGCAG-3′) and Bakt_805R (5′-GACTACHVGGGTATCTAATCC-3′) (35). Fungal diversity was assessed by amplifying the ITS2 region using primers ITS3 (5′-GCATCGATGAAGAACGCAGC-3′) and ITS4 (5′-TCCTCCGCTTATTGATATGC-3′) [34]. Sequencing reads were filtered with a quality threshold of Q30, and singletons and sequences shorter than 200 bases were removed using Cutadapt version 3.5 [35]. The data were analyzed using Mothur software version 1.44, (Ann Arbor, MI, USA) following the standard operating procedure (SOP) for Illumina MiSeq libraries [36]. Normalized data and operational taxonomic unit (OTU) assignments were based on the SILVA v138 database (Bremen, Alemania), in accordance with the methodology described in [37].

### 2.4. Physical Quality of Green Coffee Beans

The physical analysis of each sample of green coffee beans included the determination of dry matter content [38], ash content [39], and titratable acidity [40]. The percentage of healthy beans—those free from defects—was assessed by selecting them from a 450 g sample of dehulled coffee. In addition, seed viability was evaluated using the tetrazolium test, following the method described by de Freitas et al. (2020) [41].

### 2.5. Determination of Sensorial Quality of the Coffee Beverage 

Dry parchment coffee from each sampling point and treatment was dehulled to obtain green coffee beans. Specialty Coffee Association of America protocol—SCAA 2015 [42] was used for roasted, ground, and beverage preparation. For the last process, a ratio of 8.25 g of roasted coffee per 150 mL of water was used. The resulting roasted and ground coffee was evaluated by three tasters trained in sensory analysis, following the Specialty Coffee Association (SCA) protocol [42]. To obtain just one score for a sample, each taster assessed five cups per sample, scoring key attributes—fragrance/aroma, flavor, aftertaste, acidity, body, balance, overall impression, uniformity, sweetness, and clean cup—on a scale from 6 to 10. The total score (SCA points) was calculated by summing the individual scores and was used to classify the coffee. The tasters also recorded the main sensory descriptors associated with the flavor of each sample.

### 2.6. Statistical Analysis

The experiment followed a randomized complete block design (RCBD) in a 2 × 2 + 1 factorial arrangement. Three experimental blocks were made, using the quality of the raw material as the blocking factor, for a total of 15 fermentations and 99 samples corresponding to the sampling times. The mean, standard deviation, median, minimum, and maximum values were calculated for all variables. ANOVA and Repeated Measures Analysis were applied to evaluate variable behavior at each sampling point, using a significance level of 5%. All statistical analyses were performed using SAS/STAT software version 9.4 (Carolina del Norte, NC, USA) [43].

To assess the relative efficiency of the treatments across experimental units, a synthetic index was developed based on quantitative variables selected for their statistical significance. This index was constructed using principal component analysis (PCA).

## 3. Results

### 3.1. Fermentation Processes Behavior

**Figure 1:**
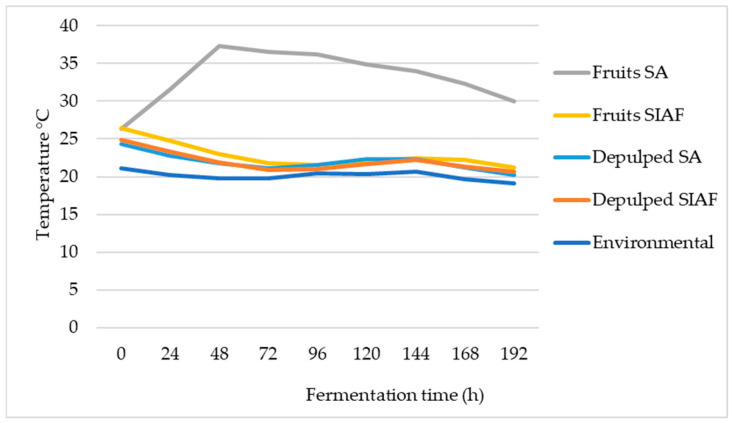
Temperature trends over time during fermentation of whole fruits and depulped coffee under two conditions: Semi-anaerobic and Self-Induced Anaerobic Fermentation (SIAF), along with ambient temperature.

Changes in the course of fermentation were reflected in the behavior over time of variables such as temperature, pH, glucose consumption, and lactic acid production. During the experiments, ambient temperature was kept steady at 20.1 ± 0.6 °C. Under these conditions, the control treatment lasted 21.2 ± 1.2 h. All fermentation processes—both semi-anaerobic (SA) and self-induced anaerobic (SIAF)—took place at temperatures above ambient, with temperature differences (ΔT) ranging from 0.5 to 3.7 °C for SIAF and from 5.1 to 17.6 °C for SA. The most notable difference occurred in the fermentation of whole fruits under semi-anaerobic conditions: from 48 h onward, the temperature stayed above 30 °C until the end of the process (Figure 1).

**Figure 2:**
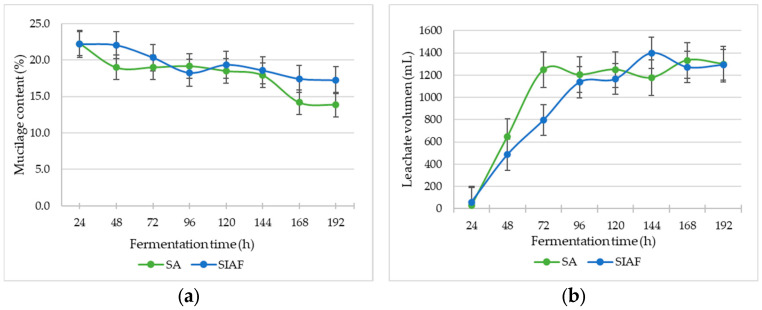
Changes over time in: (**a**) Mucilage content (%); (**b**) Leachate volume (mL) in coffee fruits fermented under two conditions: Semianaerobic (SA) and Self-Induced Anaerobic Fermentation (SIAF).

The fruits under both conditions were depulped at the established sampling times and showed a significant decrease in mucilage content as fermentation progressed (*p* < 0.0001), regardless of whether the condition was SA or SIAF (*p* = 0.2727) (Figure 2a). This trend is associated with the formation of leachates from 20 kg of coffee fruits in each bioreactor, which reached their highest production rates at 72 h under semi-anaerobic conditions (1250 mL) and at 144 h under SIAF conditions (1400 mL) (Figure 2b).

Fermentation time (*p* < 0.0001) and raw material type (pulped coffee or whole fruits) significantly influenced acidity (pH) (*p* = 0.0024), glucose content (*p* = 0.0230), and lactic acid concentration (*p* = 0.0182). Compared to pulped coffee, fermentations using whole fruits resulted in higher glucose levels and lower acidity, as indicated by both pH and lactic acid values. Notably, pH differences were significant up to 48 h of fermentation, with the SIAF condition showing reduced acidity. Similarly, fruit fermentations under SIAF had significantly higher glucose concentrations than those under the SA condition (*p* = 0.0068) (Table S1).

**Figure 3:**
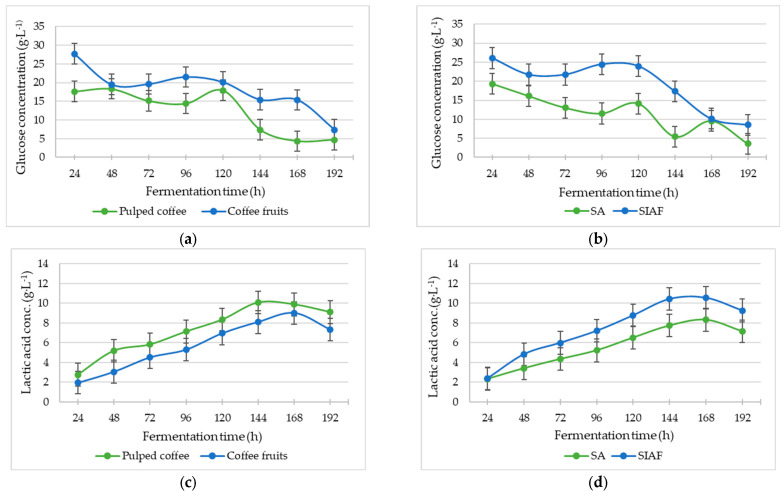
Concentrations from mucilage fermentations of: (**a**) glucose in depulped and coffee fruits; (**b**) glucose under Semi-anaerobic (SA) and Self-induced fermentation (SIAF); (**c**) Lactic acid in depulped and coffee fruits; (**d**) Lactic acid under Semi-anaerobic (SA) and Self-induced fermentation (SIAF).

The progression of fermentation, as reflected by glucose consumption (Figure 3a,b) and lactic acid production (Figure 3c,d) in the mucilage, was significantly affected by the fermentation condition (SA or SIAF), with *p =* 0.0073 and *p =* 0.0080, respectively. The highest concentrations of both compounds were recorded under the SIAF condition (Figure 3b,d).

### 3.2. Bacterial and Fungal Populations Identified

The concentration of extracted DNA ranged from 10.1 to 80.4 ng·mg^−1^, which was sufficient and representative of the samples. For bacterial diversity, read counts ranged from 106,252 to 204,678, while fungal diversity yielded between 145,822 and 290,470 reads. In total, the number of sequences ranged from 17,959 to 18,341 for 16S and from 13,077 to 13,130 for ITS. All sequences met the study’s quality criteria, with coverage analysis indicating values above 99% (Table S2).

One sample—fruits fermented under semi-anaerobic conditions at 96 h—could not be amplified for either amplicon (16S or ITS), despite meeting quality and quantity requirements based on the established filters. Similarly, three samples from fruit fermentations under SIAF at 48, 96, and 192 h showed no amplification for the ITS marker. In all three cases, DNA quality and quantity were sufficient, as successful amplification of the 16S marker confirmed, serving as a positive control. These results indicate that the samples mentioned had a very low or absent population of OTUs corresponding to the ITS region.

An average of 260 ± 71 bacterial OTUs and 101 ± 24 fungal OTUs were identified from the grouped reads (Table S3). According to the Chao1, Shannon, and Simpson indices, the control and the 48 h SIAF of pulped coffee exhibited the lowest richness, abundance, and diversity for both bacteria and fungi. In contrast, pulped coffee fermented under SA conditions for 144 h showed the highest bacterial richness, abundance, and diversity, while the highest fungal diversity was observed at 48 h (Table S3).

**Figure 4:**
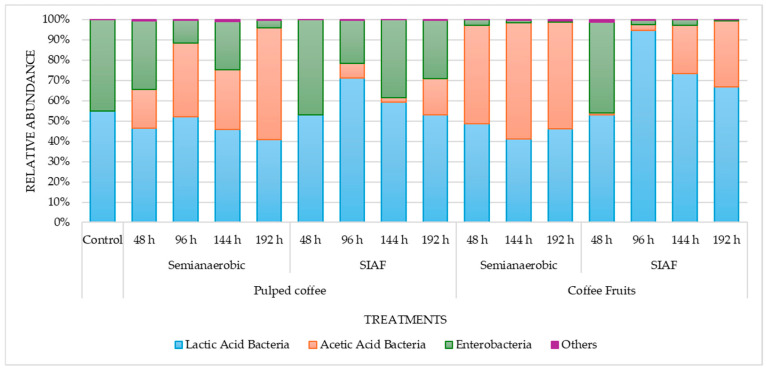
Relative abundance of the main bacterial groups—lactic acid bacteria (LAB), acetic acid bacteria (AAB), and enterobacteria—under two fermentation conditions.

Bacterial diversity was dominated by lactic acid bacteria (LAB), acetic acid bacteria (AAB), and enterobacteria, which together accounted for more than 99% of the abundance (Figure 4). Other taxa—such as *Paenibacillus*, *Herbaspirillum*, *Nocardioides*, *Curtobacterium*, *Methylobacterium*–*Methylorubrum*, and *Actinomycetospora*—originating from the rhizosphere and/or the surrounding environment, made up less than 1% of the microbiota in the fermentation processes and were therefore not considered relevant to the outcomes.

Overall, the ratio between LAB and enterobacteria populations remained similar to that of the control during the first 48 h of fermentation under SIAF conditions, in both pulped coffee and whole fruits. A significant shift was observed in the proportion of AAB, particularly in the semi-anaerobic fermentations, where they made up between 20% and 57% under these conditions.

**Figure 5:**
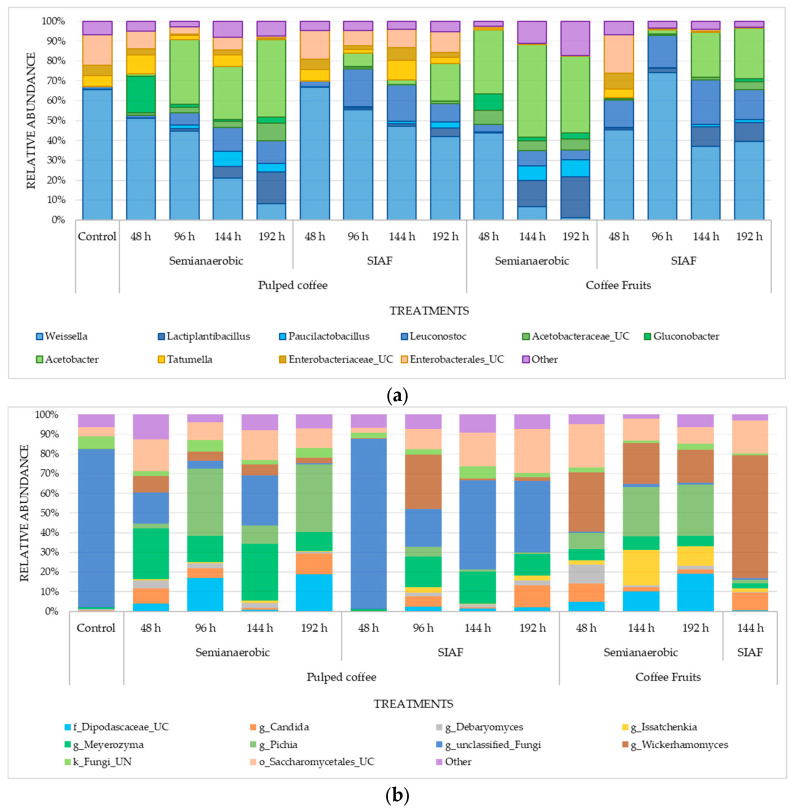
Abundance and taxonomic assignment at the genus level across different fermentation processes for (**a**) bacteria (16S); (**b**) yeast (ITS).

The most commonly identified bacterial genera (Table S5) were *Acetobacter*, *Gluconobacter*, *Leuconostoc*, *Lactiplantibacillus*, *Paucilactobacillus*, *Weissella*, *Tatumella*, along with several genera from the Enterobacteriaceae and Acetobacteraceae families (Table S4) (Figure 5a). Fermentation conditions influenced the abundance of these bacteria. In semi-anaerobic fermentations, *Weissella* decreased over time, while *Acetobacter* and *Lactiplantibacillus* became more abundant as fermentation progressed.

For the ITS region, the fungal genera identified included *Pichia*, *Issatchenkia*, *Meyerozyma*, *Candida*, *Debaryomyces*, and *Wickerhamomyces*, with their abundance varying depending on the specific fermentation conditions (Figure 5b). Numerous unclassified sequences were also found for this marker, likely due to limitations in the SILVA database (Silva.nr v138.1). Although regularly updated, the database may still lack fungal sequences from tropical environments such as the one studied here.

Genera belonging to the order Saccharomycetales were present in all fermentations, establishing themselves as a dominant group (Table S5). Members of this order are widely recognized for their abundance on coffee fruits both before and after pulping, which enhances their fermentative capacity during wet processing [44]. Notable families within this group include Pichiaceae, Saccharomycetaceae, Debaryomycetaceae, and Saccharomycodaceae, (Table S4) which encompass genera such as *Pichia*, *Candida*, *Saccharomyces*, *Meyerozyma*, and *Hanseniaspora* [45,46]—all recognized for their potential as fermentation starters.

As observed with the bacterial groups, the yeast communities in both the control sample and during the first 48 h of the SIAF process exhibited similar abundance patterns. Among the genera identified, *Pichia* was more abundant in semi-anaerobic fermentations, whereas *Issatchenkia* increased in abundance during the later stages of fruit fermentation under SA conditions. In contrast, *Wickerhamomyces*, emerged as the dominant genus in fruit fermentations conducted under SIAF (Figure 5b).

### 3.3. Physicochemical Quality of Green Coffee Beans

The physical characteristics assessed included dry matter content, ash content, acidity, and the proportion of healthy green coffee beans. The study also examined how the different fermentation processes affected embryo viability. In the initial control samples (traditional fermentation), beans showed an ash content of 3.366 ± 0.428%, titratable acidity of 2.41 ± 0.21 mg citric acid/L, and a dry matter content of 48% ± 0.01%. The proportion of healthy beans and embryo viability was 84.3% ± 10.7% and 83.3% ± 12.2%, respectively.

The use of either whole fruits or pulped coffee, combined with the chosen fermentation condition (SA or SIAF), resulted in significant changes in dry matter content, which decreased progressively with fermentation time (*p* = 0.0286), as shown in Table 1. Although changes in acidity and ash content were not statistically significant, ash content showed a tendency to increase as fermentation time lengthened.

**Figure 6:**
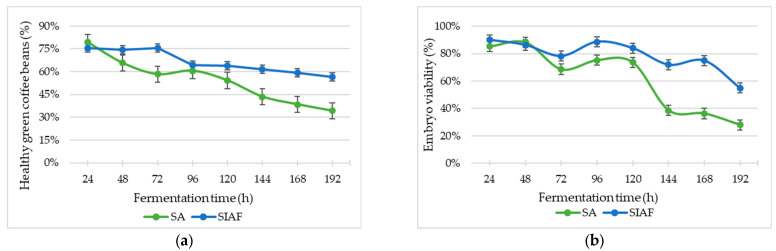
Behavior of (**a**) healthy green coffee beans and (**b**) embryo viability in coffee fermentation processes under two conditions: Semi-anaerobic (SA) and Self-Induced Anaerobic Fermentation (SIAF).

With regard to green coffee beans’ physical quality, the proportion of healthy beans was among the most affected variables across the different fermentation processes. This proportion was significantly influenced by fermentation condition—markedly lower under semi-anaerobic (SA) conditions (*p* = 0.0126)—as well as by fermentation time (*p* < 0.0001) and the interaction between these two factors (*p* < 0.0001) (Figure 6a).

Embryo viability also declined significantly over time (*p* < 0.0001). The greatest reduction was observed in samples from the SA condition (*p* = 0.0002). The interaction between fermentation type and duration also had a significant impact, with the least damage seen in whole fruits fermented under SIAF conditions, up to 120 h (*p* = 0.0043) (Figure 6b).

### 3.4. Sensorial Quality of the Coffee Beverage

The samples of coffee used for sensorial analysis from the treatments and fermentation times were qualified as specialty coffee [42], with a cupping score of 83.14 ± 0.39 SCA points. Repeated Measures ANOVA under the proposed experimental design showed no significant differences at time zero across experimental blocks (*p* = 0.1804), confirming the initial high quality of the coffee. Similarly, the treatments did not significantly impact sensory quality, regardless of raw material type (*p* = 0.6669), fermentation condition (*p* = 0.5740), fermentation time (*p* = 0.3338), or their interaction (*p* > 0.05).

Of all the samples, only one—fruit fermentation under SA at 72 h—exhibited a phenolic defect. The rest scored between 80.75 and 85.00 SCA points (Table 2).

**Figure 7:**
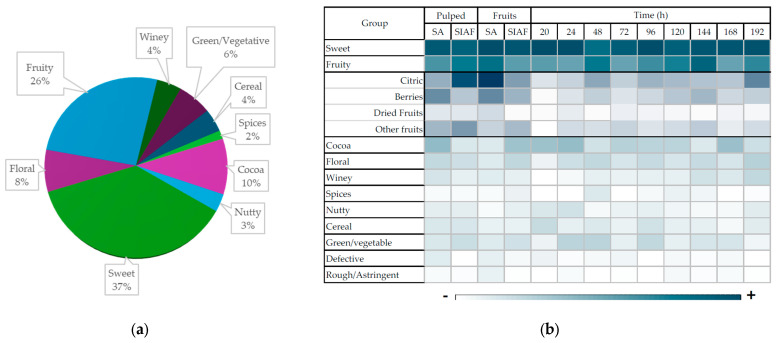
Sensory descriptors of coffee from different fermentation processes: (**a**) Frequency of descriptor groups; (**b**) heatmap showing descriptor intensity. Dark blue means a higher frequency of the descriptor; light blue means a lower frequency of the descriptor; no color means the descriptor was not presented.

Flavor and aroma descriptors were recorded for each sample and analyzed based on their frequency and intensity, then grouped into sensory categories and subcategories (Figure 7). All samples were predominantly characterized by sweet and fruity notes, typical of Colombian coffee varieties. Among the fruity descriptors, citrus and berry notes were more prominent in fruit fermentations under semi-anaerobic conditions, while tropical notes—such as yellow fruits, mango, pineapple, and peach—appeared more frequently in the SIAF system.

A quality index was developed using principal component analysis (PCA), based on the SCA sensory score and three quantitative variables that showed significant effects in the experimental design: percentage of healthy beans, embryo viability (%), and yield factor. The latter, closely related to the proportion of healthy beans, is commonly used in Colombia to assess the physical quality of coffee for commercial purposes. It indicates the number of kilograms of dry parchment coffee needed to produce 70 kg of Excelso coffee beans, the standard unit for international trade. The resulting Quality Fermentation Index (QFI) provides a comprehensive measure of both sensory and physical coffee quality, as well as overall process efficiency (time).

Principal component analysis showed that the first two components captured most of the total variance—87.73%—with PC1 contributing 62.70% and PC2 25.03%. These components were then used to construct the index. The index was calculated for each of the 99 observations, each representing a unique combination of factors at a specific fermentation time within an experimental block. Data were first normalized using Equations (1) and (2), applying the values of each component (1 or 2) for observation *i*, along with the corresponding minimum *(PCmin_i_*) and maximum (*PCmax_i_*) values. The weight of each component was then determined based on the proportion of variance it explained, as specified in Equation (3). Finally, the Fermentation Quality Index was calculated using Equation (4). Based on the resulting values, samples were classified into four categories: excellent (0.80–1.00), good (0.60–0.79), acceptable (0.40–0.59), and poor (<0.40) (Table 3).

Index for the first and second components:

(1) $${Índex\;PC}_{1i}=\frac{{PC}_{i}-{PC}_{1min}}{{PC}_{1max}-{PC}_{1min}}$$

(2) $${Índex\;PC}_{2i}=\frac{{PC}_{i}-{PC}_{2min}}{{PC}_{2max}-{PC}_{2min}}$$

(3) $${Weighted}_{PCi}=\frac{{PC}_{i\;}Value}{{PC}_{1\;}Value+{PC}_{2\;}Value}$$

(4) $$Quality\;Fermentation\;Index\;(QFI)={Index\;Pc}_{1i}\left({Weighted}_{PC1}\right)+{Índice\;Pc}_{2i}\left(1-{Weighted}_{PC1}\right)$$

The control samples were classified as excellent, driven by their higher proportion of healthy beans, greater embryo viability, superior cupping scores, and better yield factor. Overall, the number of samples rated as excellent decreased with longer fermentation times. The SIAF condition produced the highest concentration of excellent samples up to 72 h, for both pulped coffee and whole fruits—especially the latter, which consistently yielded samples rated good or excellent. The semi-anaerobic condition, in contrast, resulted in a greater number of samples classified as acceptable or poor, particularly when whole fruits were used in the fermentation process.

## 4. Discussion

This study focused on evaluating three groups of variables: those related to the progression of the fermentation process, physical and physiological quality, and sensory quality.

The course of fermentation can be effectively monitored through temperature shifts, which reflect the metabolic activity of microorganisms [47,48]. Yeasts, in particular, play a key role in converting the sugars present in the mucilage and fruit pulp into alcohols and other metabolites [7,15]. Microbial activity is strongly influenced by environmental conditions, with an optimal temperature range between 20 and 30 °C. In contrast, AAB and LAB exhibit higher activity between 15 and 25 °C [49,50]. These groups contribute to fermentation dynamics, aid in lowering pH, and serve as indicators of fermentation progress. In this study, fermentation of whole fruits under semi-anaerobic (SA) conditions exceeded this optimal range, reaching up to 37 °C at 48 h (Figure 1). Under these conditions, mucilage breakdown accelerated during the first 48 h, accompanied by rapid fruit dehydration (Figure 2), likely driven by microbial production of pectinases, a predominance of AAB (Figure 4), and a drop in pH (Table S1) due to acetic acid production. Such microbial activity has previously been linked to fermentation temperatures above 30 °C [19,47,48]. Overall, the SA condition favored microorganisms with aerobic metabolism, whose respiration generates ATP and releases heat [51,52]. In contrast, in oxygen-restricted environments such as SIAF—whether with whole fruits or pulped coffee—temperature remained near ambient (20 °C), as anaerobic metabolism prioritizes compound transformation and is less efficient at generating heat due to lower ATP production [51,52,53].

This behavior contrasts with the findings of da Mota et al., 2022 [19], who reported temperatures reaching 30.5 °C for whole fruits and 29.67 °C for pulped coffee during SIAFs lasting 87 h—attributed to the metabolic activity of yeasts and LAB. In the present study, mucilage under SIAF conditions exhibited higher concentrations of glucose and lactic acid (Figure 3b,d), which were in turn associated with increased LAB activity, particularly at 96 h (Figure 4). This pattern likely reflects the influence of lower fermentation temperatures in promoting the growth of LAB and yeasts [10,16,47].

da Silva Vale et al., 2023 and Lee et al., 2023 [16,47] also identified temperature as a key factor shaping microbial community structure, with higher fermentation temperatures favoring the dominance of Enterobacteria. The physical state of the coffee—pulped or whole—further influenced the presence of this group, with greater abundance observed in whole fruits (Figure 4). Interestingly, the similar proportions of Enterobacteria and LAB between the control and SIAFs at 48 h suggest that SIAF delays the onset of fermentation, likely due to the time required to deplete oxygen and generate CO_2_, thereby creating the anaerobic environment necessary for LAB adaptation and population growth by 96 h.

The pH remained within the normal range for this type of process [10,32,37] even with extended fermentation times. However, variations were observed depending on the state of the coffee (Table S1), suggesting that the presence of the exocarp (skin) on the fruits delays the onset of fermentation. This is reflected in the lower acidity observed at 24 h compared to pulped coffee, particularly under SIAF conditions. Similarly, lower acidity was also found in whole fruit fermentations under SA conditions, likely due to this environment promoting higher ethanol production in solid-state fermentations (10).

Within the LAB group, *Weissella* was the most abundant genus, although its prevalence varied with fermentation conditions, showing a marked decrease under SA conditions (Figure 5a). Other studies have reported an opposite trend, with *Weissella* populations increasing during longer fermentations (36 h); this variation may be due to competition among LAB genera, as *Weissella* ranked third in abundance after *Leuconostoc* and *Lactobacillus* [54]. Along with *Leuconostoc*, *Lactiplantibacillus*, and *Paucilactobacillus*; *Weissella* is considered part of the native coffee fermentation microbiota and has been shown to significantly influence beverage attributes [37,48,54,55,56], as lactic acid produced during sugar metabolism has been linked to a well-balanced coffee profile [54,57].

These bacterial genera are also known to produce bacteriocins during the logarithmic and stationary growth phases, acting as antagonistic metabolites against certain pathogenic bacteria and fungi [58,59,60]. This could explain the inhibition of some OTUs observed in ITS marker DNA sequencing, alongside environmental factors such as pH, temperature, oxygen concentration, and water activity—conditions that limit microbial growth, as reported by Jimenez et al., 2025 [28]. Higher glucose concentrations were also detected in whole fruit samples compared to pulped coffee throughout fermentation (Figure 3a), a trend expected given the complex carbohydrates in the pulp, which provide a carbon source that supports the high microbial diversity typically present [23,24]. Conversely, the lower lactic acid concentrations in whole fruit (Figure 3c) may reflect a greater balance between AAB and LAB groups under SA conditions, where higher acetic acid production likely occurred due to the heterofermentative nature of the dominant genera, which produce lactic and acetic acids, alcohols, CO_2_, and other compounds [54,60].

In solid-state fermentations (SSF), significant fruit dehydration was observed—particularly under SA conditions—likely due to elevated microbial activity. This resulted in a notable reduction in dry matter as fermentation progressed and a slightly higher, though not statistically significant, ash content (Table 1). By contrast, submerged fermentations exhibited mineral loss (ash) from the fruit as a result of leaching into the water [8,61]. Ash content is generally associated with better quality in roasted coffee and is influenced by the crop’s nutritional status, growing location, and coffee species. In *Coffea arabica* varieties, values above 5% are considered indicative of impurities [62]. Thus, the fermentation method affects ash content, making it important to consider not only the type and duration of the process but also the raw material used and whether water is added.

The physiological quality of the beans revealed a marked difference in embryo viability between the two fermentation conditions. Under SIAF, viability remained above 80% for up to 120 h. In contrast, under SA conditions, embryo deterioration began rapidly after 48 h, falling to as low as 25% by 192 h (Figure 6b). The type of raw material—whether whole fruit or depulped coffee—had no significant effect on embryo viability. Both types exhibited a decline in germination as fermentation time progressed, aligning with the trends reported by Jiménez et al., 2025 [28] for fermentation lasting up to 180 h.

This trend is likely due to the protective fruit layers surrounding the endosperm, which delay germination. In depulped coffee, germination starts on the first day of fermentation, whereas it takes longer when the exocarp remains intact, as in dry processing [63]. Prolonged fermentation and its specific conditions can affect embryo characteristics, likely due to increased microbial activity, anoxic environments, and elevated temperatures [64]. Reduced germination and damage to cell membranes have also been linked to higher lactic acid production under SIAF, along with enzymatic changes that impair the seed’s physiological quality [28,63]. Embryo viability and germination capacity are key indicators of physiological quality and are also associated with the beverage’s sensory profile [41,63], as changes in the chemical composition of the endosperm—caused by nutrient consumption during germination—can impact flavor development [28].

The decline in embryo viability is clearly linked to endosperm deterioration. Fermentation processes affected the physical quality of the beans, as evidenced by a significant reduction in the proportion of sound beans over time—particularly under SA fermentation conditions (Figure 6a). This deterioration is likely the result of a combination of semi-anaerobic conditions, prolonged fermentation, and elevated temperatures, all of which contribute to endosperm breakdown and, consequently, grain quality loss.

Previous studies have shown that although embryo viability tends to decline in yeast-assisted fermentations under SIAF conditions, cup quality is not necessarily compromised—or, conversely, that high embryo viability does not always translate into superior cup quality [41,64]. These findings are consistent with the present study: all samples across treatments were rated as specialty coffee according to SCA standards [42], with sweet and fruity notes being the most prominent overall.

However, there was a notable decline in physical quality under SA conditions for both types of raw material as fermentation time increased. Hernández-Alcántara et al., 2023 [65] reported a higher percentage of defects after 15 days of fermentation under both anaerobic methods—carbonic maceration and fermentation without CO_2_ injection (SIAF)—with the latter showing more defects and a 2.5-point drop in sensory quality compared to traditional processing.

Although physical quality remains a key criterion for buyers when assessing coffee, its relationship with fermentation processes aimed at enhancing flavor has not been extensively explored. The QFI values differentiated the samples through an integrated analysis of physical, physiological, and sensory variables, revealing that the highest overall quality was obtained under SIAF conditions—both for whole fruit and depulped coffee—when fermentation lasted less than 96 h.

## 5. Conclusions

This study aimed to assess the effects of fermentation under varying conditions defined by the coffee’s processing state, oxygen availability, and fermentation duration. Whether using whole fruits or depulped coffee, the progression of fermentation was influenced by differences in chemical composition and microbial composition, although coffee sensory quality remained unaffected. Fermentation time proved to be the most influential factor, impacting numerous variables, including fermentation dynamics, embryo viability, and physical grain quality—especially beyond 72 h. Oxygen availability played a key role in shaping the microbiota responsible for transformation and, ultimately, the final quality. Self-induced anaerobic fermentation (SIAF) notably promoted glucose consumption and lactic acid production, associated with higher LAB populations, reduced yeast activity, and greater embryo viability. It is necessary to continue exploring these types of fermentations to obtain the best potential coffee quality. The results also suggest that fermenting whole fruits under semi-anaerobic or open-tank conditions—commonly referred to as double fermentation, pre-coffee cherry, or fruit reserve—is not advisable beyond 24 h, due to its negative impact on physical quality and embryo viability. Finally, the proposed Quality Fermentation Index (QFI) offers a promising tool for evaluating coffee quality, supporting both producer income and product consistency for consumers. This index should be taken into account in future research to determine the effect of different fermentation processes on the overall quality of coffee. Additionally, complement the studies by involving variables that explain the changes in chemical composition that are related to sensory quality.

## Figures and Tables

**Table 1:** Mean values and standard deviations for dry matter, ash content, and titratable acidity of green coffee beans from fermentation processes using pulped and whole fruit coffee under semi-anaerobic and self-induced anaerobic fermentation conditions.

Coffee Stage	Fermentation		Dry Matter (%)		Ash (%)		Titratable Acidity (mg Citric Acid/L)	
	Condition	Time (h)	Average	StdDev	Average	StdDev	Average	StdDev
Pulped coffee	SA	24	48.1	0.9	3.338	0.415	2.58	0.20
		48	47.2	1.8	3.329	0.399	2.79	0.27
		72	46.9	2.5	3.374	0.344	2.59	0.52
		96	45.8	2.4	3.577	0.089	2.43	0.47
		120	44.1	6.3	3.539	0.039	2.56	0.03
		144	44.7	5.2	3.397	0.029	2.90	0.62
		168	46.6	2.9	3.570	0.316	3.04	0.06
		192	46.2	3.4	3.536	0.260	2.61	0.07
	SIAF	24	46.6	1.4	3.312	0.441	2.55	0.35
		48	47.0	2.5	3.374	0.442	2.81	0.13
		72	46.9	1.9	3.553	0.026	2.62	0.50
		96	46.8	1.1	3.408	0.193	2.41	0.01
		120	47.6	2.2	3.517	0.056	2.39	0.01
		144	46.3	1.8	3.398	0.010	2.58	0.08
		168	46.2	2.8	3.596	0.293	2.57	0.14
		192	46.2	2.8	3.648	0.148	2.45	0.30
Coffee fruits	SA	24	48.5	1.4	3.440	0.313	2.52	0.14
		48	48.0	2.2	3.369	0.292	2.82	0.20
		72	48.5	1.8	3.598	0.010	2.39	0.44
		96	47.0	0.7	3.617	0.038	2.63	0.30
		120	47.1	1.5	3.521	0.070	2.81	0.47
		144	47.5	1.4	3.491	0.101	2.42	0.20
		168	47.5	1.8	3.608	0.356	3.16	0.09
		192	47.1	1.4	3.762	0.047	2.84	0.00
	SIAF	24	48.0	0.7	3.403	0.456	2.65	0.16
		48	48.2	2.8	3.316	0.201	2.48	0.40
		72	47.8	2.0	3.623	0.071	2.46	0.34
		96	46.5	2.0	3.570	0.030	2.53	0.30
		120	47.9	2.2	3.484	0.024	2.33	0.38
		144	48.3	2.6	3.744	0.276	2.54	0.03
		168	47.8	1.5	3.683	0.215	2.46	0.26
		192	47.0	1.5	3.793	0.101	2.51	0.32

**Table 2:** Descriptive analysis of coffee quality (SCA points) under different fermentation conditions.

Coffee Stage	Fermentation		(SCA Points)			
	Condition	Time (h)	Average	Min	Max	StdDev
Pulped coffee	SA	24	81.83	81.67	82.00	0.17
		48	83.39	83.25	83.50	0.13
		72	81.67	80.75	83.17	1.31
		96	83.36	82.33	84.75	1.25
		120	82.64	82.25	83.00	0.38
		144	83.92	83.75	84.17	0.22
		168	83.42	83.25	83.50	0.14
		192	83.56	82.75	84.00	0.70
	SIAF	24	83.22	82.33	84.00	0.84
		48	82.69	81.42	83.75	1.18
		72	83.22	82.75	83.67	0.46
		96	82.94	82.75	83.17	0.21
		120	83.50	82.50	84.08	0.87
		144	82.81	82.25	83.33	0.54
		168	83.00	82.17	84.42	1.23
		192	83.39	83.00	83.75	0.38
Coffee fruits	SA	24	83.03	82.50	83.75	0.65
		48	83.47	83.17	83.75	0.29
		72	83.92	83.67	84.17	0.35
		96	84.11	83.25	85.00	0.88
		120	83.47	82.83	83.83	0.55
		144	84.19	83.67	85.00	0.71
		168	83.06	82.92	83.17	0.13
		192	84.11	83.75	84.50	0.38
	SIAF	24	83.33	83.25	83.50	0.14
		48	83.36	83.08	83.83	0.41
		72	83.53	82.67	84.00	0.75
		96	82.92	82.17	83.42	0.66
		120	83.39	83.00	83.58	0.34
		144	82.92	82.33	83.42	0.55
		168	82.72	82.33	83.17	0.42
		192	83.39	82.75	83.92	0.59

**Table 3:** Average and Standard Deviation for QFI and heatmap for coffee samples classification; P: Poor, A: Acceptable, G: Good and E: Excellent. Dark green indicates a greater number of samples in the classification; light green indicates a smaller number of samples in the classification; no color indicates that there were no samples in that classification.

Coffee Stage	Fermentation		Index ICF		Classification			
	Condition	Time (h)	Average	StdDev	P	A	G	E
Pulped coffee	SA	24	0.887	0.044				
		48	0.822	0.084				
		72	0.783	0.090				
		96	0.816	0.046				
		120	0.755	0.094				
		144	0.671	0.107				
		168	0.561	0.066				
		192	0.497	0.172				
	SIAF	24	0.887	0.043				
		48	0.851	0.046				
		72	0.871	0.010				
		96	0.789	0.036				
		120	0.815	0.046				
		144	0.771	0.028				
		168	0.728	0.060				
		192	0.662	0.063				
Coffee Fruits	SA	24	0.887	0.020				
		48	0.822	0.048				
		72	0.685	0.180				
		96	0.718	0.080				
		120	0.673	0.015				
		144	0.430	0.132				
		168	0.457	0.027				
		192	0.369	0.081				
	SIAF	24	0.880	0.049				
		48	0.882	0.021				
		72	0.848	0.062				
		96	0.834	0.071				
		120	0.791	0.090				
		144	0.745	0.085				
		168	0.772	0.093				
		192	0.730	0.078				

## Data Availability

Data are contained within the article or Supplementary Material. High-throughput sequencing (HTS) data are available in repository NCBI SRA BioProject, accession no: PRJNA1254155.

## Supplementary Materials

The following supporting information can be downloaded at: https://www.mdpi.com/article/10.3390/foods14173001/s1, Table S1: Average values and standard deviation to pH, glucose and lactic acid concentrations (g·L^−1^), in different fermentation processes, Table S2: Concentration of DNA of samples from different fermentation processes and sequencing quality from DNA extracted, Table S3: Results of richness and diversity bacterial 16S and fungal ITS region by Illumina MiSeq amplicon sequencing, Table S4: Relative abundance of bacterial and fungal family level obtained by metataxonomic analysis from 16S and ITS rRNA gene sequences, for samples coffee in semi-anaerobic (SA) and self-induced anaerobic (SIAF) fermentation processes, and different conditions such as pulped coffee and coffee fruit from 24 h to 192 h, Table S5: Relative abundance of bacterial and fungal genera level obtained by metataxonomic analysis from 16S and ITS rRNA gene sequences, for samples coffee in semi-anaerobic (SA) and self-induced anaerobic (SIAF) fermentation processes, and different conditions such as pulped coffee and coffee fruit from 24 h to 192 h.
