# Supplementary material for: Development of Fermentation Strategies for Quality Mild Coffee Production (Coffea arabica L.) Based on Oxygen Availability and Processing Time
Source: Foods. 2025 Aug 27;14(17):3001. doi: 10.3390/foods14173001 (PMC12427761; doi:10.3390/foods14173001)
Supplement: Supplementary file 1 [file foods-14-03001-s001.zip › Suplementary_material_Tables S1-S2-S3.pdf]

Table S1. Average values and standard deviation to pH, glucose and lactic acid concentrations (g·L^{-1}), in different fermentation processes.

**Fermentation conditions**	**Sampling time (h)**	**pH**		**Glucose content (g·L^{-1})**		**Lactic Acid Content (g·L^{-1})**	
		**Average**	**StdDev**	**Average**	**StdDev**	**Average**	**StdDev**
Control treatment	Beginning	5.38	0.18	67.68	94.02	N.D.	
	Ending	3.98	0.24	25.91	2.76	2.55	1.37
Pulped coffee Semianaerobic	24	4.08	0.02	19.07	6.08	2.95	0.56
	48	3.64	0.33	15.61	3.79	4.27	1.05
	72	3.68	0.21	12.83	8.77	5.24	1.04
	96	3.70	0.02	16.66	1.98	6.75	0.58
	120	3.63	0.03	19.78	22.50	7.40	1.72
	144	3.64	0.06	6.86	11.43	9.88	0.20
	168	3.61	0.03	5.17	7.53	8.27	3.11
	192	3.59	0.09	4.38	6.63	9.55	2.24
Pulped coffee SIAF	24	4.27	0.43	16.22	4.98	2.57	1.11
	48	3.90	0.03	21.05	4.39	6.10	0.78
	72	3.83	0.05	17.45	6.42	6.41	0.51
	96	3.78	0.12	12.15	9.75	7.52	1.73
	120	3.70	0.01	16.11	17.64	9.29	2.78
	144	3.60	0.05	7.89	12.89	10.28	2.21
	168	3.63	0.05	3.51	4.97	11.50	2.70
	192	3.60	0.05	5.06	6.68	8.66	2.29
Coffee Fruits Semianaerobic	24	4.24	0.16	19.55	4.83	1.70	0.58
	48	3.87	0.07	16.58	3.82	2.57	1.34
	72	3.86	0.18	13.19	2.69	3.46	1.32
	96	3.89	0.17	6.37	1.82	3.71	1.69
	120	3.91	0.17	8.48	10.52	5.64	0.41
	144	4.08	0.35	4.03	3.71	5.62	0.19
	168	3.92	0.17	13.96	16.50	8.40	6.02
	192	4.05	0.28	2.76	1.81	4.79	0.44
Coffee Fruits SIAF	24	4.81	0.09	35.91	21.97	2.21	0.64
	48	4.38	0.06	22.42	6.79	3.57	1.47
	72	4.24	0.02	26.02	4.63	5.58	1.60
	96	4.08	0.03	36.68	30.48	6.89	1.13
	120	3.99	0.02	31.79	22.59	8.26	0.87
	144	3.90	0.03	26.81	8.66	10.58	2.77
	168	3.85	0.07	16.79	2.90	9.61	1.68
	192	3.83	0.08	12.02	11.43	9.87	1.12

N.D. Not Detected

Table S2. Concentration of DNA of samples from different fermentation processes and sequencing quality from DNA extracted.

Coffee stage	Fermentation		Concentration (ng/µL)	16S				ITS			
	Condition	time (h)		Raw reads	Q30	nseqs	coverage	Raw reads	Q30	nseqs	coverage
	Control treatment		22,8	133,686	83.3	18,298	0.99	229,300	86.0	13,087	0,99
Pulped coffee	SA	48	40,0	157,228	83.5	18,299	0.99	218,762	85.7	13,085	0,99
		96	80,4	138,776	83.3	18,310	0.99	145,822	82.8	13,130	0,99
		144	12,6	106,252	81.3	18,281	0.99	180,682	80.0	13,081	0,99
		192	31,1	130,784	84.3	18,246	0.99	213,518	85.8	13,093	0,99
	SIAF	48	29,4	182,148	84.1	18,318	0.99	180,682	80.0	13,081	0,99
		96	34,6	146,296	84.2	18,310	0.99	189,700	82.0	13,098	0,99
		144	12,7	145,524	84.2	18,297	0.99	179,100	73.1	13,119	0,99
		192	14,1	148,274	82.3	18,279	0.99	218,746	73.6	13,098	0,99
Coffee fruits	SA	48	13,8	168,152	84.9	17,959	0.99	290,470	88.5	77,141	0,99
		96	26,1	---	---	---	---	---	---	---	---
		144	44,9	158,012	84.0	18,304	0.99	224,858	85.6	13,081	0,99
		192	12,8	144,666	84.0	18,313	0.99	229,300	86.0	13,087	0,99
	SIAF	48	40,0	173,400	82.2	18,314	0.99	---	---	---	---
		96	10,1	160,274	84.0	18,298	0.99	---	---	---	---
		144	22,5	130,480	85.2	18,341	0.99	225,292	80.1	13,077	0,99
		192	29,1	204,678	85.1	17,983	0.99	---	---	---	---

--- : No data available for quality from DNA extracted due to the DNA from the sample not amplifying for 16S and/or ITS regions

Table S3. Results of richness and diversity bacterial 16S and fungal ITS region by Illumina MiSeq amplicon sequencing.

Coffee stage	Fermentation		16S					ITS				
	**Condition**	**time (h)**	**Observed**	**Chao1**	**ACE**	**Shannon**	**Simpson**	**Observed**	**Chao1**	**ACE**	**Shannon**	**Simpson**
Control			193	492	415	1.74	0.68	164	300	291	1.19	0.36
Pulped coffee	SA	48	264	444	504	2.29	0.83	100	445	209	2.63	0.87
		96	295	633	716	2.08	0.77	84	150	135	2.39	0.83
		144	394	527	569	2.75	0.88	83	163	155	2.33	0.83
		192	261	913	712	2.18	0.73	82	169	138	2.32	0.82
	SIAF	48	124	194	192	1.70	0.71	102	128	141	0.98	0.35
		96	297	606	702	2.26	0.83	135	342	371	2.57	0.87
		144	233	489	593	2.20	0.82	104	153	181	1.98	0.74
		192	309	919	704	2.30	0.83	84	134	138	2.51	0.86
Coffee fruits	SA	48	173	320	328	1.80	0.74	88	147	161	2.40	0.84
		96	---	---	---	---	---	---	---	---	---	---
		144	360	594	681	2.07	0.79	72	87	93	2.22	0.84
		192	253	495	434	2.15	0.79	111	217	224	2.32	0.85
	SIAF	48	332	731	609	2.40	0.84	---	---	---	---	---
		96	269	446	478	1.75	0.77	---	---	---	---	---
		144	251	566	517	2.16	0.81	102	173	164	1.79	0.67
		192	152	248	289	2.00	0.82	---	---	---	---	---

--- : No data available for richness and abundance indices due to the DNA from the sample not amplifying for 16S and/or ITS regions.
